# Public policy coverage and access to medicines in Brazil

**DOI:** 10.11606/s1518-8787.2022056003898

**Published:** 2022-06-20

**Authors:** Ricardo Montes de Moraes, Maria Angelica Borges dos Santos, Fabiola Sulpino Vieira, Rosimary Terezinha de Almeida

**Affiliations:** I Instituto Brasileiro de Geografia e Estatística Rio de Janeiro RJ Brasil Instituto Brasileiro de Geografia e Estatística. Rio de Janeiro, RJ, Brasil; II Universidade Federal do Rio de Janeiro Instituto Alberto Luiz Coimbra de Pós-Graduação e Pesquisa em Engenharia Programa de Engenharia Biomédica Rio de Janeiro RJ Brasil Universidade Federal do Rio de Janeiro.Instituto Alberto Luiz Coimbra de Pós-Graduação e Pesquisa em Engenharia. Programa de Engenharia Biomédica. Rio de Janeiro, RJ, Brasil; III Fundação Oswaldo Cruz Escola Nacional de Saúde Pública Rio de Janeiro RJ Brasil Fundação Oswaldo Cruz.Escola Nacional de Saúde Pública. Rio de Janeiro, RJ, Brasil; IV Instituto de Pesquisa Econômica Aplicada Brasília DF Brasil Instituto de Pesquisa Econômica Aplicada. Brasília, DF, Brasil

**Keywords:** Health Services Accessibility, National Policy of Pharmaceutical Assistance, Drugs of Continuous Use, Drugs, Essential, Program Evaluation

## Abstract

**OBJECTIVE:**

Describe consumption patterns for monetary and non-monetary acquisition of medicines according to age and income groups, highlighting pharmaceuticals associated with health programs with specific access guarantees.

**METHODS:**

Descriptive observational study using microdata from the 2017–2018 *Pesquisa de Orçamentos Familiares* (Household Budget Survey, POF/IBGE). We initially reviewed programs/policies with specific guarantees of access to medicines in the SUS. Using the pharmaceutical product list of POF-4 (chart 29 of the questionnaire on individual expenditures), we selected the medicines related to these programs. We then described frequencies and percentages for not reporting medicine consumption and for reporting consumption (either through monetary or non-monetary acquisition) according to age and income groups. For medicines with distinctive access guarantees, we compared average monthly values of acquisitions and consumption patterns by age and income.

**RESULTS:**

63% of those in the ≤ 2 minimum wage (MW) household income group did not report consuming medicines in the last month. Among those earning > 25 MW, 44.3% did not report consumption. Non-monetary acquisitions of medicines were mainly reported for the < 10 MW group and for the elderly and accounted for 20.5% of the total consumption of medicines (in value). For policies with specific access guarantees, non-monetary acquisitions reached 33.6% of total consumption. This percentage varied for the various selected medicines: vaccines, 83.3%; cancer drugs, 70.3%; diabetes, 47.9%; hypertension, 35.9%; asthma and bronchitis, 29.2%; eye problems, 14%; prostate and urinary tract, 10.7%; gynecological, 11.6%; and contraceptives, 9.7%.

**CONCLUSION:**

Shares for non-monetary acquisitions of medicines are still low but benefit mainly lower-income and older age groups. Policies and programs with specific access guarantees to medicines have increased access. Results suggest the need to strengthen and expand pharmaceutical care policies.

## INTRODUCTION

Pharmaceuticals are a significant component of the global health spending. Among Organization for Economic Cooperation and Development (OECD) member countries, they account for 20% of total health expenditures^1^. In Brazil, they account for 18.4% of the country´s expenditures on health goods and services, and for 29.2% of household health expenditures^2^, especially compromising the most vulnerable^3^. This highlights the importance of public funding in access to medicines.

Public policies on pharmaceutical coverage are defined according to their breadth, scope, and depth (whether copayment is required to obtain medicines)^4^. Pharmaceutical market regulatory and pricing policies^3,5^ determine expenditures incurred by households and, ultimately, their access to medicines.

The breadth of pharmaceutical coverage defines the percentage of the population having access via public funding. OECD countries usually provide comprehensive coverage for medicines through government reimbursement schemes or specific insurance schemes^6^. Restrictions concern the scope of medicines available (positive and negative lists for government funding)^7^and whether copayment is required^6^. Emerging countries, on the other hand, do not provide full public coverage. In practice, they restrict public funding to specific demographic or population segments or diseases, with a limited scope of medicines available^4^.

In Brazil, the *Política Nacional de Medicamentos* (PNM – National Medicine Policy) and the *Política Nacional de Assistência Farmacêutica* (PNAF – National Pharmaceutical Care Policy), issued in 1998 and 2004, respectively, established guidelines and strategic axes to secure access to medicines and to promote their rational use. They also defined the *Relação Nacional de Medicamentos Essenciais* (Rename – National List of Essential Medicines)^8,9^. As of 2012, the Rename lost its role in guiding public supply of medicines and started being considered a positive list for public funding by the three spheres of government^10^.

In addition to the expectation of securing access to medicines in the Rename, the Brazilian National Health System (SUS) has organized its pharmaceutical care (PC) around several programs and policies targeting specific population segments or diseases. These include, in variably explicit ways, distinctive guarantees of access to medicines. Mapping the policies that provide these distinctive guarantees is a good starting point to monitor performance in this area.

Nationwide studies on the extent of pharmaceutical coverage in Brazil are scarce. Data on public procurement may be obtained from government administrative records and used to produce information on the availability of medicines. There are significant gaps in these data, notably regarding purchases by states and municipalities. In addition, data lack information on the scope and population coverage by the PC policies.

The *Pesquisa de Orçamentos Familiares* (POF – Household Budget Survey)^11^is an infrequently used source that may allow us to obtain more details on coverage by type of medicine, beneficiary and consumption (divided into monetary and non-monetary acquisitions). In addition to reporting out of pocket (OOP) expenditures on pharmaceuticals (monetary acquisitions), the POF asks respondents to estimate the monetary values of medicines obtained as non-monetary acquisitions. This provides potential information on public funding for medicines. The POF also allows us to describe the distribution of consumption by income and age groups, both for monetary and non-monetary acquisitions.

Starting by systematizing SUS programs and policies containing explicit PC guarantees, we sought to identify patterns of coverage and consumption for non-monetary acquisition by income and age groups. Understanding who benefits from non-monetary acquisitions is crucial to monitor and evaluate policy results in this area.

## METHODS

This is an observational study using data from the *Pesquisa de Orçamentos Familiares* (POF) of the *Instituto Brasileiro de Geografia e Estatística* (IBGE – Brazilian Institute of Geography and Statistics). We initially reviewed the Health Legislation for laws, ordinances and norms related to health programs specifically including the provision of medicines among their objectives. We then went on to identify the main current specific guarantees of access to pharmaceutical coverage in the SUS.

The POF is a household survey with a sample of 57,920 households selected in conglomerates at the different strata of the survey. It has national representativeness and aims to describe the consumption and income patterns, as well as the living conditions of Brazilian households. Using the variables in chart 29 of the POF’s individual expenditure questionnaire (Questionnaire 4), comprising 88 types of pharmaceutical products, we prepared translators to associate each of the previously identified programs with the corresponding POF’s types of pharmaceutical products. Two authors - a physician and a pharmacist - selected and matched the pharmaceutical product types to the related PC programs. Both authors were seasoned in public health management.

We used the latest edition of the POF, covering from July 11, 2017 to July 9, 2018. Data on pharmaceutical products in the survey refer to those obtained in the 30 days preceding the interview.

The survey provides data on consumption expenditures and mode of acquisition (variable *V9002*), divided into ‘monetary acquisition’ and ‘non-monetary acquisition’ (without OOP payment by those obtaining the medicine). For the non-monetary acquisitions, respondents also report their estimated values for the medicines obtained. The POF microdata also include deflated values for the January 2018 reference period (variable *V8000_defla*) to prevent price variations over the data collection period from distorting the interpretation of results^11^.

For each type of pharmaceutical product listed in the POF, the number of people reporting consumption and the reported values for monetary and non-monetary acquisitions were aggregated according to age (*V0403*) and income groups (variable *Renda_total (Total_household_income)* for those obtaining the medicine).

Aggregation by age groups covers 14 age ranges from 0–19 years-old to > 80 years-old, at five or ten-year intervals. Aggregations by income include seven ranges: ≤ two minimum wages (MW), 2–3 MW, 3–6 MW, 6–10 MW, 10–15 MW, 15–25 MW, and > 25 MW.

To check the consistency between values estimated by respondents for non-monetary acquisitions and those informed for monetary acquisitions (proxy of market prices), we calculated the average monthly values for monetary and non-monetary acquisitions for each type of pharmaceutical product.

A descriptive analysis, with one-off and interval-based estimates, was carried out for: (a) average monthly values and percentages of subjects reporting consumption; (b) total consumption of medicines; (c) percentage of subjects reporting non-monetary acquisitions among those obtaining medicines from programs with specific guarantees of access.

Variables with small frequencies in the sample were excluded from tables. Analyses were performed using the *R* software (version 4.0.3), and the *survey* package (version 4.0), which considers the sampling design of the survey. The share of subjects reporting non-monetary acquisition in total consumption was described by age and income groups. We used the *svyciprop* function to calculate 95% confidence intervals^12^.

## RESULTS

Brazil has several health programs or policies involving specific guarantees of access to medicines. In addition to the PNM and the PNAF, which are more comprehensive, we identified those policies and programs and related them to the specific types of pharmaceutical products surveyed in the POF (Box).

**Box:** Specific guarantees of access to medicines in health policies and programs, according to POF types of pharmaceutical products.

Policies/Programs	Specific guarantees of access to medicines	POF pharmaceutical product type
*Política Nacional de Atenção em Oftalmologia* (National Eye Care Policy)	Pharmaceutical Care in SUS as a policy issue, focusing on glaucoma treatment. Medicines included in the *Componente Especializado da Assistência Farmacêutica* (CEAF – Specialized Pharmaceutical Care Component)^3,14^.	For eye disorders (ophthalmology)
*Política Nacional de Atenção Integral à Saúde da Mulher* (National Policy for Women’s Healthcare)	Provision of contraceptive methods to the fertile population. It includes oral and injectable contraceptives, intrauterine device (IUD), and diaphragm^13,14^.	Contraceptive
		For gynecological disorder
*Política Nacional para a Prevenção e Controle do Câncer na Rede de Atenção à Saúde das Pessoas com Doenças Crônicas* (National Policy for Cancer Prevention and Control in the Health Care Network for Chronic Diseases)	Access to chemotherapy. With the emergenceof oral chemotherapy drugs, the high complexity oncology centers (*Centros de Alta Complexidade em Oncologia*, CACON) and the high complexity oncology care units (*Unidades de Assistência em Alta Complexidade em Oncologia*, UNACON) started dispensing chemotherapy drugs for use by patients at home. The Ministry of Health performs centralized purchase of some antineoplastic drugs aiming to reduce the cost of treatment in SUS, and increase the population’s access to treatment^13,15^.	For cancer
*Política de Saúde Mental* (Mental Health Policy)	Access to medicines for mental suffering or disorder. The *Relação Nacional de Medicamentos Essenciais* (Rename – National List of Essential Medicines) includes medicines for the treatment of several health conditions in this field: anxiolytics, antidepressants, and antipsychotics, among others^13,14,16^.	For autism
		For depression (antidepressant)
		For stress (tranquilizer)
		For the nervous system
*Política Nacional de Transplantes de Órgãos e Tecidos* (National Policy on Organ and Tissue Transplant)	There is no formal policy with this denomination There is, however, a National Transplant System, in addition to clinical protocols and therapeutic guidelines that establish the use of immunosuppressants and other drugs in the treatment of transplanted patients. Medicines listed in the protocols are part of the Rename^14,17^.	Immunosuppressants
*Política Nacional de Atenção Integral da Saúde do Homem* (National Policy for Men’s Healthcare)	Its guidelines include, among others, the treatment of male diseases and illnesses. The Rename includes drugs for the treatment of benign prostatic hyperplasia^13,14^.	For prostate and urinary tract
*Programa Farmácia Popular do Brasil* (Popular Pharmacy Program in Brazil)	Dispensing by private pharmacies, upon users’ co-payment, of a list of drugs used to treat dyslipidemia, osteoporosis, glaucoma, rhinitis, and Parkinson’s disease, as well as contraceptives, and geriatric diapers. Free dispensing to the patient of Programa Farmácia Popular do Brasil (Popular Pharmacy Program in Brazil) used in the treatment of hypertension, diabetes, and asthma^18,19^.	For asthma and bronchitis
		For diabetes
		For bones and joints
		For high blood pressure (antihypertensive)
		For lowering cholesterol or triglycerides
*Programa Nacional de Controle do Tabagismo* (Brazilian Tobacco Control Program)	Access to medicines to combat smoking^14,16^.	Combat alcoholism and smoking
*Programa Nacional de DST/aids* (National STDs/Aids Program)	Access to antiretroviral drugs, and other drugs for treatment of opportunistic diseases^14,20^.	For Aids
		Condom and intimate lubricant
*Programa Nacional de Imunizações* (National Immunization Program)	Access to vaccines and serums^14,21^.	Vaccines
*Programas Estratégicos de Saúde* (Strategic Health Programs)	There is no formal program with this denomination. It includes a set of health programs, such as control of tuberculosis, leprosy, focal endemics, flu (influenza), as well as prevention of nutritional deficiencies, and the blood and blood products program^14,22^.	For infectious and endemic diseases


POF: Pesquisa de Orçamentos Familiares; STDs: sexually transmitted diseases.

When considering the universe of pharmaceutical products with data collected in the POF (and not just those with specific guarantees of access in the SUS), 63% of the ≤ 2 MWs household income group did not report obtaining medicines in the last month. In the > 25 MWs group, this percentage was 44.3% (Figure). Consumption percentages decrease along income groups, suggesting budgetary restrictions to consumption in lower income groups and/or excessive consumption in higher income groups.

**Figure:**
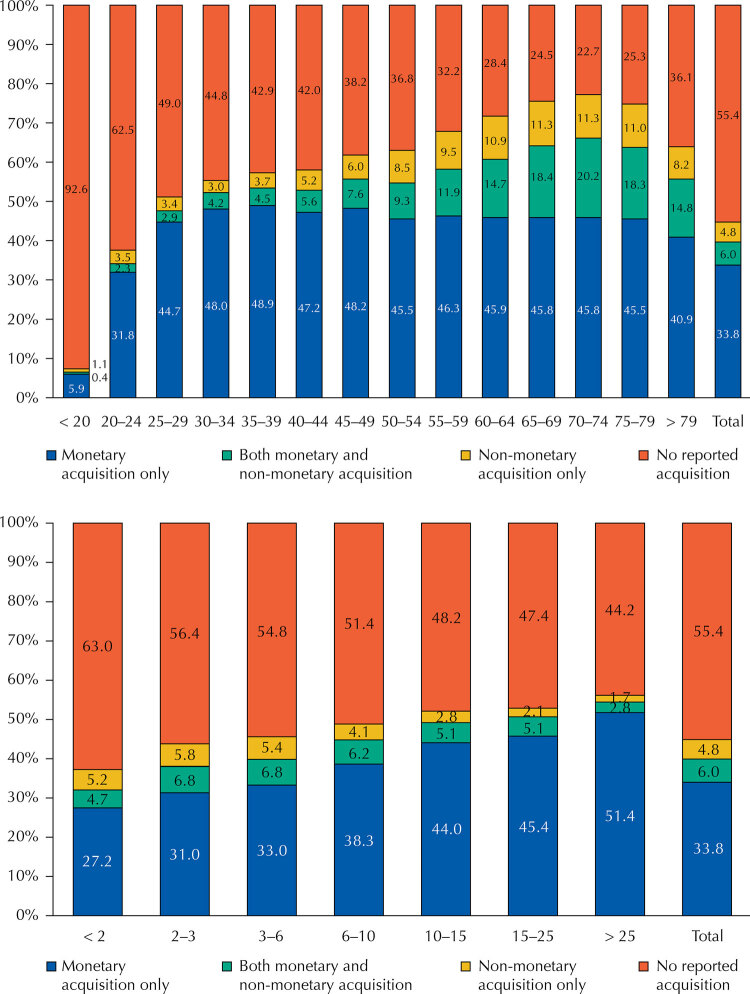
Mode of acquisition of medicines in the last 30-days (% in total consumption) according to age and income groups. Brazil, 2017–2018.

Source: data from the *Pesquisa de Orçamentos Familiares* (POF), 2017–2018.

Subjects earning < 10 MWs have the greatest shares of non-monetary acquisitions. Nevertheless, shares for non-monetary acquisitions are low across all income groups, varying from 5.8% to 1.7% for exclusively non-monetary acquisition (Figure).

Starting from the 2–3 MWs group, non-monetary acquisitions - including the ‘both monetary and non-monetary acquisition’ category depicted in Figure - decrease as income increases. The lowest share for this compound non-monetary acquisitions is seen in the > 25 MWs group (4.5%). For the ≤ 2 MWs group, this percentage is 9.9%.

In the analysis by age group, non-monetary acquisition of medicines is higher among the elderly, where most subjects regularly consume medicines. Also, 39.2% of the non-monetary acquisitions are concentrated in the > 60 year-old groups. In the 70–74 years-old age group, 77.3% reported monetary and/or non-monetary consumption of medicines in the month prior to the survey.

Considering both monetary and non-monetary acquisitions recorded in the POF, medicines related to specific PC programs accounted for 41.4% of the total consumption value, but concentrate 67.8% of the non-monetary acquisitions. Among all medicines with specific guarantees of access with reported consumption, 33.6% were non-monetary acquisitions. In other words, even though most of the medicines obtained by non-monetary means are related to PC programs, non-monetary acquisitions represent only one-third of their total consumption.

Over half of pharmaceutical products listed in the POF (58.6%) are not related to any health program or policy. In this group, 11.3% of the consumption value refers to non-monetary acquisitions.

Table 1 presents average monthly values for monetary and non-monetary acquisitions for the selected access-related POF pharmaceutical products, in addition to monetary acquisitions (value and subjects) as a share of total consumption. For hypertension, cholesterol, prostate, and urinary tract pharmaceuticals, the average monthly value of monetary acquisitions was higher than for non-monetary acquisitions. For eye conditions the opposite occurred, with a higher average value for non-monetary acquisitions. For the other products, price differences (average value per purchase) were within the confidence intervals.

**Table 1:** Average monthly values (R$) for monetary and non-monetary acquisitions and share (95%CI) of monetary acquisitions (values and persons) in total expenditure for selected pharmaceuticals with SUS specific policy-related guarantees.

Pharmaceutical product	Average monthly monetary acquisition (R$)	Average monthly non-monetary acquisition (R$)	Monetary acquisitions as a share (%) of total expenditure	Subjects (%) reporting monetary acquisition
Vaccines	181.9 (89.5–274.3)	143.2 (109.0–177.4)	16.7 (6.5–26.9)	13.6 (7.4–19.8)
For cancer	234.7 (160.5–308.8)	307.4 (174.6–440.2)	29.7 (17.1–42.4)	36.2 (24.9–47.5)
For diabetes	96.1 (89.5–102.8)	70.9 (64.1–77.6)	52.1 (48.5–55.7)	45.8 (43.9–47.7)
For infectious and endemic diseases	147.1 (124.3–169.9)	207.2 (162.6–251.8)	58.5 (46.9–70.0)	69.5 (60.6–78.4)
For high blood pressure (antihypertensive)	47.9 (46.3–49.6)	34.8 (33.3–36.3)	64.1 (62.4–65.9)	57.7 (56.4–59.0)
For lowering cholesterol or triglycerides	48.6 (46.2–51.0)	39.3 (35.1–43.5)	68.7 (65.2–72.2)	64.7 (62.3–67.0)
For the nervous system	117.9 (109.3–126.5)	116.8 (102.0–131.6)	70.0 (66.1–73.8)	72.6 (70.2–75.0)
For asthma and bronchitis	93.5 (84.1–103.0)	98.8 (84.2–113.5)	70.8 (64.5–77.1)	74.1 (68.9–79.4)
For depression (antidepressant)	110.7 (103.3–118.1)	95.2 (83.4–106.9)	74.3 (70.9–77.8)	72.6 (70.3–74.9)
Condom and intimate gels	15.6 (10.3–21.0)	13.8 (11.8–15.9)	75.3 (67.5–83.1)	73.3 (68.8–77.8)
For stress (tranquilizer)	59.9 (56.2–63.7)	52.5 (44.0–60.9)	77.4 (74.0–80.9)	76.0 (73.6–78.4)
For bones and joints	97.8 (90.0–105.6)	93.4 (79.5–107.3)	81.8 (78.0–85.6)	82.3 (79.3–85.2)
For eye disorders (ophthalmology)	65.2 (60.7–69.9)	89.6 (73.0–106.2)	86.0 (83.0–89.1)	90.7 (89.1–92.2)
For gynecological disorder	58.6 (51.7–65.5)	54.3 (29.4–79.2)	88.4 (82.1–94.7)	87.6 (84.4–90.9)
For prostate and urinary tract	85.5 (78.8–92.2)	57.8 (46.2–69.5)	89.3 (85.6–93.0)	85.0 (80.6–89.4)
Contraceptive	23.5 (22.6–24.5)	20.2 (18.4–21.9)	90.3 (89.0–91.6)	89.0 (87.8–90.1)

Source: data from the *Pesquisa de Orçamentos Familiares* (POF), 2017–2018.

95%CI: confidence interval of 95%; SUS: *Sistema Único de Saúde* (National Health System).

Note: medications with small sample size or non-significant results were not included.

Ranking medicines according to monetary acquisitions as a percentage of the total consumption evidences a relationship between this percentage and the average monthly value per purchase. The share of monetary acquisitions is usually higher for the lower- priced medicines.

Considering expenditures (values) reported for all the pharmaceutical products listed in the POF, 20.5% referred to non-monetary acquisitions. Although most of the pharmaceutical consumption in Brazil relies on out of pocket payment (OOP) by households (monetary acquisition), there are items which are mainly obtained by non-monetary means. This is the case of vaccines (83.3% not depending on OOP). Table 2 shows the estimated values for monthly acquisitions and the number of subjects obtaining medicines through monetary and non-monetary acquisition according to policy and POF type of pharmaceutical product.

**Table 2:** Monthly monetary and non-monetary consumption of medicines with specific guarantees provided, in amount (R$ million) and number of subjects obtaining them (in thousands) (95%CI).

Policies and programs / POF pharmaceutical products	Monetary acquisitions (million R$)	Non-monetary acquisitions (million R$)	Monetary acquisitions (in thousand inhabitants)	Non- monetary acquisitions (in thousand inhabitants)
*Política Nacional de Atenção em Oftalmologia* (National Eye Care Policy)	**168.2 (151.7–184.7)**	**27.4 (20.9–33.8)**	**2,578.4 (2,397.8–2,759.0)**	**305.4 (250.3–360.6)**
For eye disorders (ophthalmology)	168.2 (151.7–184.7)	27.4 (20.9–33.8)	2,578.4 (2,397.8–2,759.0)	305.4 (250.3–360.6)
*Política Nacional de Atenção Integral à Saúde da Mulher* (National Policy for Women’s Healthcare)	**192.0 (179.9–204.1)**	**21.6 (17.9–25.3)**	**7,138.0 (6,792.0–7,484.0)**	**910.2 (816.4–1,003.9)**
Contraceptive	153.9 (143.8–164.1)	16.6 (14.3–18.9)	6,543.2 (6,213.9–6,872.4)	821.4 (730.5–912.3)
For gynecological disorder	38.1 (31.8–44.3)	5.0 (2.1–8.0)	650.1 (573.2–727.0)	92.5 (66.8–118.1)
*Política Nacional de Atenção Oncológica* (Brazilian Policy on Oncology Care)	**18.8 (10.5–27.1)**	**44.4 (25.9–63.0)**	**80.1 (54.8–105.6)**	**144.6 (92.8–196.3)**
For cancer	18.8 (10.5–27.1)	44.4 (25.9–63.0)	80.1 (54.8–105.5)	144.6 (92.8–196.3)
*Política Nacional de Saúde Mental* (Brazilian Mental Health Policy)	825.5 (777.0–874.1)	299.3 (268.0–330.6)	7,858.0 (7,527.0–8,190.0)	2,982.9 (2,792.5–3,173.4)
For depression (antidepressant)	331.1 (300.5–361.7)	114.3 (96.7–132.0)	2,991.4 (2,801.1–3,181.6)	1,201.3 (1,091.3–1,311.3)
For stress (tranquilizer)	194.1 (176.7–211.6)	56.6 (45.3–67.8)	3,239.2 (3,038.9–3,439.6)	1,078.1 (957.9–1,198.4)
For the nervous system	299.1 (270.5–327.6)	128.4 (107.1–149.7)	2,537.3 (2,364.2–2,710.5)	1,099.6 (984.4–1,214.7)
*Política Nacional de Atenção Integral da Saúde do Homem* (National Policy for Men’s Healthcare)	**48.9 (42.1–55.8)**	**5.9 (3.8–8.0)**	**572.2 (501.5–642.9)**	**101.5 (69.0–134.0)**
For prostate and urinary tract	48.9 (42.1–55.8)	5.9 (3.8–8.0)	572.2 (501.5–642.9)	101.5 (69.0–134.0)
*Programa Farmácia Popular do Brasil* (Popular Pharmacy Program in Brazil)	**1,336.3 (1,275.0–1,397.5)**	**796.1 (748.2–844.0)**	**18,374.0 (17,841.0–18,908.0)**	**13,233.0 (12,775.5–13,690.5)**
For asthma and bronchitis	60.8 (51.2–70.4)	25.1 (18.6–31.5)	650 (569.4–730.5)	253.5 (195.2–311.8)
For diabetes	328.5 (298–359.1)	302.2 (268.4–336.0)	3,418 (3,225.7–3,610.3)	4,264 (4,043.3–4,484.7)
For bones and joints	123.2 (107.6–138.7)	27.5 (21.2–33.7)	1,260 (1,139–1,381)	294.1 (243.2–345)
For high blood pressure (antihypertensive)	639.8 (609.5–670.1)	357.7 (336.2–379.2)	13,352.7 (12,923.5–13,781.9)	10,277.5 (9,874.6–10,680.4)
For lowering cholesterol or triglycerides	184 (168.9–199.1)	83.7 (71.5–95.9)	3,782.8 (3,541.5–4,024.2)	2,129.3 (1,960.2–2,298.5)
*Programa Nacional de DST/aids* (National STDs/Aids Program)	**13.6 (8.6–18.6)**	**56.4 (24.8–88.0)**	**873.4 (740.4–1,006.5)**	**360.0 (293.9–426.1)**
Condom and intimate lubricant	13.6 (8.6–18.6)	4.5 (3.5–5.4)	873.4 (740.4–1,006.5)	323.3 (260.8–385.8)
*Programa Nacional de Imunizações* (National Immunization Program)	**8.8 (3.0–14.5)**	**43.8 (28.8–58.7)**	**48.2 (24.6–71.8)**	**305.6 (231.3–379.9)**
Vaccines	8.8 (3.0–14.5)	43.8 (28.8–58.7)	48.2 (24.6–71.8)	305.6 (231.3–379.9)
*Programas Estratégicos de Saúde* (Strategic Health Programs)	**29.6 (21.4–37.8)**	**21.1 (12.3–29.8)**	**201.3 (152.0–250.7)**	**101.6 (67.7–135.5)**
For infectious and endemic diseases	29.6 (21.4–37.8)	21.1 (12.3–29.8)	201.3 (152.0–250.7)	101.6 (67.7–135.5)

Source: data from the *Pesquisa de Orçamentos Familiares* (POF), 2017–2018.

95%CI: confidence interval of 95%; STDs: sexually transmitted diseases.

Note: medicines and programs with small sample size or non-significant results were not included.

In addition to vaccines, cancer drugs were also mainly obtained by non-monetary means (70.3%). Two of the three classes of medicines for chronic diseases dispensed in SUS units and by the *Programa Farmácia Popular* (Popular Pharmacy Program) stand out: medicines for diabetes and for hypertension. The POF does not specifically discriminate data for asthma medicines, another item in the *Programa Farmácia Popular* for which no copayment is required. However, medicines for “asthma and bronchitis” in the POF have 29.2% of their values in non-monetary acquisitions.

The POF did not report monetary acquisitions for Aids medicines, as they are not sold at commercial pharmacies. Thus, there is no price reference for respondents in their value estimates of non-monetary acquisitions. Small samples reporting consumption of medicines for Aids, autism, alcoholism, smoking and immunosuppressants preclude a robust estimate of monthly averages for these pharmaceuticals.

Non-monetary acquisitions also had significant shares in medicines for: infectious or endemic diseases (41.5%); cholesterol-lowering (31.3%); and nervous system (30%). Contraceptives (9.7%); prostate and urinary tract medicines (10.7%); medicines for gynecological problems (11.6%); and for eye conditions (14%) hold the smallest shares for non-monetary acquisitions in total consumption.

Table 3 shows the percentages of non-monetary acquisitions for medicines related to each health program or policy by age and income groups.

**Table 3:** Non-monetary acquisitions as shares (%) of consumption for medicines with specific guarantees of access, according to policy or program, age and income (in minimum wages) groups (95%CI).

Age groups	*Programa Nacional de DST/aids* (National STDs/Aids Program)	*Programa Farmácia Popular no Brasil* (Popular Pharmacy Program in Brazil)	*Programa Nacional de Imunizações* (National Immunization Program)	*Política Nacional de Atenção em Oftalmologia* (National Eye Care Policy)	*Política Nacional de Saúde Mental* (Brazilian Mental Health Policy)	*Política Nacional de Atenção Integral à Saúde da Mulher* (National Policy for Women’s Healthcare)
< 20	41.3 (28.2–55.7)	38.5 (26.6–51.9)	82.9 (61.9–93.5)	18.6 (7.5–39.1)	36.8 (28.4–46.1)	14.3 (10.9–18.5)
20–29	30.6 (23.6–38.6)	29.9 (24.2–36.2)	81.8 (63.0–92.2)	7.7 (3.2–17.1)	28.9 (23.0–35.5)	11.4 (9.7–13.4)
30–39	20.9 (15.0–28.3)	33.7 (30.1–37.6)	88.6 (74.4–95.4)	8.9 (4.5–16.7)	25.2 (21.7–29.0)	10.7 (8.9–12.8)
40–49	23.3 (14.8–34.7)	41.8 (39.3–44.3)	83.9 (64.1–93.8)	5.9 (3.3–10.2)	29.9 (26.6–33.5)	9.8 (7.5–12.7)
50–59	55.2 (33.4–75.2)	47.3 (45.2–49.3)	79.3 (54.2–92.6)	6.8 (4.5–10.3)	31.5 (28.5–34.6)	15.2 (9.7–23.1)
60–69	54.6 (22.9–82.9)	48.8 (46.9–50.8)	95.2 (69.5–99.4)	15.2 (10.9–20.7)	29.5 (25.9–33.3)	9.8 (2.7–30.1)
70–79	9.7 (0.2–82.8)	48.9 (46.6–51.2)	100.0 (NA)	15.4 (11.0–21.0)	26.6 (22.5–31.2)	25.1 (5.5–65.8)
> 79	NA	41.8 (38.3–45.4)	100.0 (NA)	9.5 (4.9–17.4)	18.9 (14.1–24.9)	22.0 (3.2–70.9)
**Income groups**	***Programa Nacional de DST/aids* (National STDs/Aids Program)**	***Programa Farmácia Popular no Brasil* (Popular Pharmacy Program in Brazil)**	***Programa Nacional de Imunizações* (National Immunization Program)**	***Política Nacional de Atenção em Oftalmologia* (National Eye Care Policy)**	***Política Nacional de Saúde Mental* (Brazilian Mental Health Policy)**	***Política Nacional de Atenção Integral à Saúde da Mulher* (National Policy for Women’s Healthcare)**
< 2	44.3 (34.2–54.9)	50.0 (47.8–52.1)	100.0 (NA)	15.1 (10.6–21.1)	33.8 (30.4–37.4)	16.0 (13.4–19.0)
2–3	34.6 (25.9–44.4)	50.7 (48.9–52.5)	94.5 (84.6–98.1)	13.2 (10.2–17.0)	34.9 (32.3–37.6)	12.1 (10.2–14.2)
3–6	31.7 (21.4–44.0)	51.5 (49.2–53.8)	88.6 (70.1–96.2)	11.4 (7.9–16.2)	36.4 (32.9–40.1)	15.4 (12.5–18.8)
6–10	13.6 (4.9–32.9)	30.7 (27.4–34.2)	74.3 (39.0–92.9)	4.5 (1.5–12.6)	12.4 (8.9–16.9)	5.6 (3.5–9.1)
10–15	20.4 (13.2–30.2)	43.3 (40.6–46.1)	82.8 (55.8–94.8)	8.3 (5.1–13.2)	23.4 (20.0–27.2)	8.1 (6.2–10.6)
15–25	8.4 (2.7–23.0)	24.9 (20.9–29.4)	52.1 (28.7–74.7)	4.1 (1.3–12.1)	9.8 (6.4–14.6)	5.7 (2.5–12.5)
> 25	6.5 (1.3–26.4)	13.5 (10.2–17.8)	65.8 (30.3–89.5)	3.8 (0.5–22.2)	4.4 (1.9–9.7)	4.1 (1.3–12.4)

Source: data from the *Pesquisa de Orçamentos Familiares* (POF), 2017–2018.

95%CI: confidence interval of 95%; NA: not available; STDs: sexually transmitted diseases.

Note: health programs with small sample size or non-significant results were not included.

For most programs and policies with specific medicine guarantees, differences between age groups were not significant. Sample size did not allow disaggregation by age and income in some cases.

Income groups in the 0-15 MWs range reported higher percentages of non-monetary acquisitions for the programs studied, with the exception of medicines related to the *Política Nacional de Atenção em Oftalmologia* (National Eye Care Policy), with a confidence interval too wide to support this conclusion. Households with incomes > 15 MWs showed lower shares of non-monetary acquisitions for medicines (Table 3).

## DISCUSSION

Non-monetary acquisition by households accounted for 20.5% of the total value of pharmaceutical consumption in the POF. The highest shares for non-monetary acquisitions were reported by households in the < 10 MW income groups and for older age groups. The share of public funding in pharmaceutical consumption is notoriously low in Brazil, far below the OECD average of 58%^23^.

In the *Conta-Satélite de Saúde* (Brazilian Health Satellite Accounts)^2^, an IBGE publication with aggregate data for health-related goods and services, non-monetary medicine consumption by Brazilian households represented 7.5% of total pharmaceutical consumption. The main reason for the difference in non-monetary consumption between the POF and the *Conta-Satélite* concerns prices and the type of pharmaceutical purchased by the government. The government provides medicines based on medical prescriptions and rational use. It also buys in larger scale and pays lower prices comparatively to households, as it uses the *Preços Máximos de Venda Governamental* (Government Maximum Sales Prices), which are below market prices in some cases. Thus, with equal resources, the government will buy more units of pharmaceuticals than households. On assuming similar prices for monetary and non-monetary acquisitions, one could say that the POF’s percentage for non-monetary acquisitions provides a fairer description of shares in terms of physical quantities than values obtained from the *Conta-Satélite*^24^.

The distinctive access guarantees contained in some specific health policies and programs (such as the *Programa Nacional de Imunizações* [Brazilian Immunization Program] and in the *Programa Farmácia Popular* [Popular Pharmacy Program]) have apparently secured greater non-monetary access than the list of general guarantees in the PNAF. Brazil seems thus to evidence similarities to other developing countries, which tend to concentrate public funding for PC in specific niches of populations or diseases^4^.

Even specific guarantees of access to medicines may lead to very different coverages. Among the medicines with the highest shares of non-monetary acquisition, vaccines (83.3%), and cancer drugs (70.3%) stand out. On the other end, contraceptives (9.7%), medicines for prostate and the urinary tract (10.7%), for gynecological problems (11.6%), and for eye conditions (14%) show low percentages of non-monetary obtention.

Non-monetary access to medicines for hypertension and diabetes via the *Programa Farmácia Popular* prevented hospitalizations in the SUS, and deaths related to these diseases in the municipalities hosting the program^25^. The free availability of medicines in *Programa Farmácia Popular* and the end of copayment increased their use. This suggests that, for many people, price is a barrier to access, even if products are dispensed in pharmacies of the SUS health units^26,27^.

Lower income groups obtain medicines by non-monetary means more often than higher income groups. As price is a more frequent barrier to access^26^ for them, obtaining medicines at no cost prevents the worsening of health status, potential hospitalizations and early death due to interruptions in treatment of previously diagnosed chronic diseases^25^.

This implies that the decrease in public funding for medicines reported in administrative records since 2016 may have grave effects both on population health and on expenditure on services, overloading the hospital network with preventable cases^25^.

Women’s care was the PC-sponsored program with lowest coverage. This was a surprising finding considering the prominence of this policy segment, related to goals 3 and 5 of the Sustainable Development Goals (SDG)^28^. Policies on men’s health care, eye care, and tobacco control also recorded low shares for non-monetary acquisition of medicines. Among the more recent policies, OOP values required to buy some medications with low prices in private pharmacies, such as contraceptives, may be one of the reasons for the low coverage seen for these policies. High prices and low barriers to access in SUS probably act as incentives for seeking medicines through public provision. Complexity in procedures to obtain these medicines may discourage this demand, specially burdening those with lower income. This suggests a need to simplify the procedures for medicine obtention for products reporting low shares of non-monetary acquisitions, as in the case of contraceptives.

The good coverage for the so-called ‘poverty medicines’ (vaccines and endemic diseases) draws attention. These products focus on communicable diseases and imply the oldest specific guarantees in PC, as the *Programa Nacional de Imunizações* preceded the PNAF^29^.

This study has some limitations. For the very advanced ages, small sample size leads to frail estimates, given data variability. For less-used medicines (such as for smoking or cancer), small samples produce large variances preventing meaningful analyses by age or income groups.

There are also differences between POF results for non-monetary acquisition of medicines and government administrative records for expenditures in specific programs, such as for Aids. This limitation of the database reflects the estimation of non-monetary acquisition values by POF respondents and the small sample sizes for medicines used by a small part of the population.

The survey does not record medicine acquisition by under-10-year-olds. It also reports acquisition and not actual use of the medication. So one can presume that the acquisitions made for use of younger age groups are largely made by their parents and recorded as parental acquisitions. This leads to potential overestimation of consumption for groups in charge of children or minors.

The distribution of free samples of medicines^30^ and, since 2014, the provision of oral antineoplastics and medicines for chemotherapy side effects by health plans^31^need to be acknowledged, as they may account for part of the non-monetary acquisitions. It would be thus incorrect to suppose that all non-monetary medicine acquisitions in the POF reflect SUS funding.

Estimation of values for non-monetary acquisitions by POF respondents has generated some mistrust regarding data, and could be considered a limitation. However, on comparing average monthly values for monetary and non-monetary acquisitions of the selected pharmaceuticals in our study no substantial differences were found. This strengthens the case for the use of these data. The use of POF non-monetary acquisition data is one of the positive contributions of this article to the study of PC coverage.

The policies and programs highlighted in this study provide specific medicines to their beneficiaries. It should be remembered, however, that the right to comprehensive therapeutic care is an integral part of the right to health. To ensure it, central and sub-national governments in Brazil have implemented several measures to strengthen PC in the SUS. A large network of public pharmacies is responsible for delivering PC throughout the country. However, the low availability of medicines in these pharmacies^32^ may partly explain the high percentage of monetary acquisitions for highly prevalent diseases, even when additional guarantees defined in specific policies and programs are in place. This suggests the existence of barriers to access to these products in the SUS.

The results of this article suggest the need to strengthen and expand PC policies. They have mainly benefited groups with lower income or higher age-related consumption. Data on monetary and non-monetary medicine acquisitions provided in the POF, albeit indirectly, help to describe the scope of these policies regarding access to medications.

## References

[1] Belloni A, Morgan D, Paris V. Pharmaceutical expenditure and policies: past trends and future challenges. Paris (FR): OECD; 2016. (OECD Health Working Papers; nº 87). 10.1787/5jm0q1f4cdq7-en

[2] Instituto Brasileiro de Geografia e Estatística. Conta-satélite de saúde: Brasil: 2010-2017. Rio de Janeiro: IBGE; 2019 [cited 2021 Feb 17]. Available from: http://informe.ensp.fiocruz.br/assets/anexos/6c3e434126a948bd2b5aec4eede17f92ed6ac3c8.PDF

[3] Boing AC, Bertoldi AD, Posenato LG, Peres KG. Influência dos gastos em saúde no empobrecimento de domicílios no Brasil. RevSaude Publica. 2014;48(5):797-807. 10.1590/S0034-8910.2014048005113 PMC4211571 25372171

[4] Maniadakis N, Kourlaba G, Shen J, Holtorf A. Comprehensive taxonomy and worldwide trends in pharmaceutical policies in relation to country income status. BMC Health Serv Res. 2017;17:371. 10.1186/s12913-017-2304-2 PMC5445358 28545440

[5] Dias LLS, Santos MAB, Pinto CDBS. Regulação contemporânea de preços de medicamentos no Brasil - uma análise crítica. Saude Debate;43(121):543-58. 10.1590/0103-1104201912120

[6] Vogler S, Haasis MA, Dedet G, Lam J, Pedersen HB. Medicines reimbursement policies in Europe. Copenhaguen (DK): WHO Regional Office for Europe; 2018 [cited 2021 May 21]. Available from: https://www.euro.who.int/__data/assets/pdf_file/0011/376625/pharmaceutical-reimbursement-eng.pdf

[7] Barnieh L, Clement F, Harris A, Blom M, Donaldson C, Klarenbach S, et al. A systematic review of cost-sharing strategies used within publicly-funded drug plans in member countries of the Organisation for Economic Co-Operation and Development. PLoS One. 2014;9(3):e90434. 10.1371/journal.pone.0090434 PMC3949707 24618721

[8] Ministério da Saúde (BR). Portaria Nº 3.916, de 30 de outubro de 1998. Aprova a Política Nacional de Medicamentos. Brasília, DF; 1998 [cited 2021 Feb 17]. Available from: https://bvsms.saude.gov.br/bvs/saudelegis/gm/1998/prt3916_30_10_1998.html

[9] Ministério da Saúde (BR), Conselho Nacional de Saúde. Resolução Nº 338, de 6 de maio de 2004. Aprova a Política Nacional de Assistência Farmacêutica. Brasília, DF: CNS; 2004 [cited 2021 Feb 17]. Available from: https://bvsms.saude.gov.br/bvs/saudelegis/cns/2004/res0338_06_05_2004.html

[10] Santos-Pinto CDB, Ventura M, Pepe VLE, Osorio-de-Castro CGS. Novos delineamentos da Assistência Farmacêutica frente à regulamentação da Lei Orgânica da Saúde. CadSaude Publica. 2013;29(6):1056-8. 10.1590/S0102-311X2013000600002

[11] Instituto Brasileiro de Geografia e Estatística, Diretoria de Pesquisas, Coordenação de Trabalho e Rendimento. Pesquisa de Orçamentos Familiares: primeiros resultados: 2017-2018. Rio de Janeiro: IBGE; 2019 [cited 2021 Feb 17]. Available from: https://biblioteca.ibge.gov.br/visualizacao/livros/liv101670.pdf

[12] Lumley T. The Analysis of Complex Survey Samples. Wien (AT): CRAN; 2020 [cited 2021 May 21]. Available from: https://cran.r-project.org/web//packages/survey/survey.pdf

[13] Ministério da Saúde (BR). Portaria de Consolidação Nº 2, de 28 de setembro de 2017. Consolidação das normas sobre as políticas nacionais de saúde do Sistema Único de Saúde. Brasília, DF; 2017 [cited 2021 Mar 11]. Available from: http://www.cvs.saude.sp.gov.br/zip/U_PRC-MS-GM-2_280917.pdf

[14] Ministério da Saúde (BR),Secretaria de Ciência, Tecnologia, Inovação e Insumos Estratégicos em Saúde, Departamento de Assistência Farmacêutica e Insumos Estratégicos em Saúde. Relação Nacional de Medicamentos Essenciais 2020. Brasília, DF; 2020 [cited 2021 Mar 11]. Available from: https://bvsms.saude.gov.br/bvs/publicacoes/relacao_medicamentos_rename_2020.pdf

[15] Ministério da Saúde (BR), Secretaria de Atenção à Saúde, Departamento de Regulação, Avaliação e Controle, Coordenação-Geral de Sistemas de Informação. Manual de bases técnicas da oncologia - SIA/SUS. 21. ed. Brasília, DF; 2015. [cited 2021 Mar 11]. Available from: https://portalarquivos.saude.gov.br/images/pdf/2015/outubro/16/Manual-Oncologia-21-edi----o-14-09-2015.pdf

[16] Ministério da Saúde (BR). Portaria de Consolidação Nº 3, de 28 de setembro de 2017. Consolidação das normas sobre as redes do Sistema Único de Saúde. Brasília, DF; 2017 [cited 2021 Mar 11]. Available from: https://bvsms.saude.gov.br/bvs/saudelegis/gm/2017/prc0003_03_10_2017.html

[17] Ministério da Saúde (BR). Portaria de Consolidação Nº 4, de 28 de setembro de 2017. Consolidação das normas sobre os sistemas e os subsistemas do Sistema Único de Saúde. Brasília, DF; 2017 [cited 2021 Mar 11]. Available from: https://bvsms.saude.gov.br/bvs/saudelegis/gm/2017/MatrizesConsolidacao/Matriz-4-Sistemas.html

[18] Brasil. Decreto Nº 5.090, de 20 de maio de 2004. Regulamenta a Lei no 10.858, de 13 de abril de 2004, e institui o programa “Farmácia Popular do Brasil”, e dá outras providências. Brasília, DF; 2004 [cited 2021 Mar 11]. Available from: http://www.planalto.gov.br/ccivil_03/_ato2004-2006/2004/decreto/d5090.htm

[19] Ministério da Saúde (BR). Farmácia Popular. Brasília, DF; 2021 [cited 2021 Mar 11]. Available from: https://antigo.saude.gov.br/acoes-e-programas/farmacia-popular

[20] Brasil. Lei Nº 9.313, de 13 de novembro de 1996. Dispõe sobre a distribuição gratuita de medicamentos aos portadores do HIV e doentes de AIDS. Brasília, DF; 1996 [cited 2021 Mar 11]. Available from: https://www.planalto.gov.br/ccivil_03/leis/l9313.htm

[21] Ministério da Saúde (BR). Programa Nacional de Imunizações: 30 anos. Brasília, DF; 2003 [cited 2021 Mar 11]. (Série C. Projetos e Programas e Relatórios). Available from: https://bvsms.saude.gov.br/bvs/publicacoes/livro_30_anos_pni.pdf

[22] Secretaria de Estado da Saúde do Espírito Santo. Programas do Componente Estratégico. Vitória; SD [cited 2021 Mar 11]. Available from: https://farmaciacidada.es.gov.br/programas-do-componente-estrategico

[23] Organisation for Economic Co-operation and Development. Health at a glance: OECD indicators 2019. Paris (FR): OECD; 2019. 10.1787/4dd50c09-en

[24] Vieira FS, Santos MAB. O setor farmacêutico no Brasil sob as lentes da conta-satélite de saúde. Brasília, DF: Ipea; 2020 [cited 2021 May 6]. (Texto para Discussão; nº 2615). Available from: http://repositorio.ipea.gov.br/bitstream/11058/10328/1/td_2615.pdf

[25] Almeida ATC, Sá EB, Vieira FS, Benevides RPS. Impacts of a Brazilian pharmaceutical program on the health of chronic patients. RevSaude Publica. 2019;53: 20. 10.11606/S1518-8787.2019053000733 PMC6390690 30726501

[26] Emmerick ICM, Campos MR, Luiza VL, Chaves LA, Bertoldi AD, Ross-Degnan D. Retrospective interrupted time series examining hypertension and diabetes medicines usage following changes in patient cost sharing in the ‘Farmácia Popular’ programme in Brazil. BMJ Open. 2017;7(11):e017308. 10.1136/bmjopen-2017-017308 PMC5695305 29101135

[27] Almeida ATC, Vieira FS. Copagamento dos usuários no programa Farmácia Popular do Brasil: um estudo exploratório da rede conveniada. Brasília, DF: Ipea; 2020 [cited 2021 Feb 17]. (Texto para Discussão; nº 2585). Available from: http://repositorio.ipea.gov.br/bitstream/11058/10218/1/td_2585.pdf

[28] World Health Organization. Sustainable Development Goals (SDGs). Geneva (CH): WHO; c2021 [cited 2021 May 21]. Available from: https://www.who.int/health-topics/sustainable-development-goals#tab=tab_3

[29] Santana RS, Lupatini EO, Leite SN. Registro e incorporação de tecnologias no SUS: barreiras de acesso a medicamentos para doenças da pobreza? CiencSaudeColetiva. 2017;22(5):1417-28. 10.1590/1413-81232017225.32762016 28538914

[30] Ministério da Saúde (BR), Agência Nacional de Vigilância Sanitária. Resolução-RDC Nº 60, de 26 de novembro de 2009. Dispõe sobre a produção, dispensação e controle de amostras grátis de medicamentos e dá outras providências. Brasília, DF: Anvisa; 2009 [cited 2021 May 21]. Available from: https://bvsms.saude.gov.br/bvs/saudelegis/anvisa/2009/rdc0060_26_11_2009.html

[31] Brasil. Lei Nº 12.880 de 13 de novembro de 2013. Altera a Lei nº 9656 de 3 de junho de 1998, que dispõe sobre os planos e seguros privados de “assistência à saúde”, para incluir tratamentos entre as coberturas obrigatórias. Brasília, DF; 2013 [cited 2021 May 21]. Available from: https://presrepublica.jusbrasil.com.br/legislacao/112108327/lei-12880-13

[32] Nascimento RCRM, Álvares J, Guerra Junior AA, Gomes IC, Costa EA, Leite SN, et al. Disponibilidade de medicamentos essenciais na atenção primária do Sistema Único de Saúde. RevSaude Publica. 2017; 51 Supl 2:10s. 10.11606/s1518-8787.2017051007062

