# Supplementary material for: Public policy coverage and access to medicines in Brazil
Source: Rev Saude Publica. 2022 Jun 20;56:58. doi: 10.11606/s1518-8787.2022056003898 (PMC9239427; doi:10.11606/s1518-8787.2022056003898)
Supplement: Translation

# Translation: Cobertura de políticas públicas e acesso a medicamentos no Brasil

**Authors:** Ricardo Montes de Moraes, Maria Angelica Borges dos Santos, Fabiola Sulpino Vieira, Rosimary Terezinha de Almeida

**Affiliations:** I Brasil Instituto Brasileiro de Geografia e Estatística. Rio de Janeiro, RJ, Brasil; II Brasil Universidade Federal do Rio de Janeiro. Instituto Alberto Luiz Coimbra de Pós-Graduação e Pesquisa em Engenharia. Programa de Engenharia Biomédica. Rio de Janeiro, RJ, Brasil; III Brasil Fundação Oswaldo Cruz. Escola Nacional de Saúde Pública. Rio de Janeiro, RJ, Brasil; IV Brasil Instituto de Pesquisa Econômica Aplicada. Brasília, DF, Brasil

**Keywords:** Acesso aos Serviços de Saúde, Política Nacional de Assistência Farmacêutica, Medicamentos de Uso Contínuo, Medicamentos Essenciais, Avaliação de Programas e Projetos de Saúde

## Abstract

**OBJETIVO:**

Descrever padrões de consumo de medicamentos com e sem desembolso direto de recursos, segundo faixas de idade e de renda, destacando os fármacos associados a programas de saúde com garantias específicas de acesso.

**MÉTODOS:**

Estudo descritivo observacional usando microdados da *Pesquisa de Orçamentos Familiares* (POF/IBGE) 2017–2018. Inicialmente, foram definidas as garantias específicas de acesso a medicamentos do SUS pela sistematização de programas/políticas com essa previsão. A partir dos medicamentos do quadro 29 do questionário de despesas individuais (POF-4), foram selecionados tipos de medicamentos associados a essas garantias. Foram descritas as frequências e os percentuais de pessoas sem consumo e com consumo (aquisição com e sem desembolso direto de recursos), segundo faixas de idade e de renda. Para medicamentos vinculados a garantias específicas, comparou-se valores médios mensais de aquisições e padrões de consumo por faixa etária e renda.

**RESULTADOS:**

Entre as pessoas com renda domiciliar até dois salários mínimos, 63% não declararam consumo de medicamentos no mês. Entre as acima de 25 salários mínimos, foram 44,3%. Aquisições sem desembolso direto foram feitas principalmente por pessoas na faixa com até 10 salários mínimos e entre os mais idosos. O consumo sem desembolso direto representou 20,5% do consumo total de medicamentos (em valor). Para políticas com garantias específicas de acesso, o consumo sem desembolso foi de 33,6% com variações desse percentual entre os medicamentos selecionados no estudo: vacinas, 83,3%; medicamentos para câncer, 70,3%; diabetes, 47,9%; hipertensão, 35,9%; asma e bronquite, 29,2%; problemas oftalmológicos, 14%; medicamentos para próstata e vias urinárias, 10,7%; para problemas ginecológicos, 11,6%; e anticoncepcionais, 9,7%.

**CONCLUSÃO:**

O consumo sem desembolso ainda é baixo, mas beneficia principalmente pessoas de menor renda e idosos. Políticas e programas com garantias específicas de acesso a medicamentos têm aumentado esse acesso. Os resultados sugerem a necessidade de fortalecer e ampliar as políticas de assistência farmacêutica.

## INTRODUÇÃO

Medicamentos são um componente expressivo dos gastos em saúde no mundo. Em países da Organização para a Cooperação e Desenvolvimento Econômico (OCDE), eles respondem por 20% do total dos gastos em saúde^1^. No Brasil, representam 18,4% das despesas com bens e serviços de saúde e 29,2% dos gastos das famílias com saúde^2^, onerando especialmente as mais vulneráveis^3^. Essa realidade realça a importância do financiamento público do acesso a medicamentos.

As políticas públicas de cobertura farmacêutica são definidas segundo sua amplitude (*breadth*), seu escopo (*scope*) e a exigência ou não de copagamento (*depth*) para obtenção do medicamento^4^. Com as políticas de regulação e precificação do mercado farmacêutico^3,5^, essas políticas determinam as despesas incorridas pelas famílias e, em última instância, seu acesso a medicamentos.

A amplitude da cobertura farmacêutica define o percentual da população com acesso a medicamentos via financiamento público. Países da OCDE em geral disponibilizam amplitude plena de cobertura para medicamentos mediante esquemas de reembolso governamental ou de seguros específicos^6^. As restrições dizem respeito ao escopo de medicamentos disponibilizado (listas positivas e negativas de medicamentos para financiamento governamental)^7^e à exigência ou não de copagamento^6^. Países emergentes, por outro lado, não disponibilizam cobertura pública plena. Na prática, restringem o financiamento público a segmentos demográficos ou populacionais específicos ou a algumas doenças determinadas, com um escopo reduzido de medicamentos^4^.

No Brasil, a Política Nacional de Medicamentos (PNM) e a Política Nacional de Assistência Farmacêutica (PNAF), aprovadas em 1998 e 2004 respectivamente, estabeleceram, entre suas diretrizes e eixos estratégicos, a garantia do acesso da população a medicamentos e a promoção do seu uso racional. Definiram ainda como lista orientadora da oferta pública, a Relação Nacional de Medicamentos Essenciais (Rename)^8,9^. A partir de 2012, a Rename perdeu seu caráter norteador e passou a ser tratada como uma lista positiva para financiamento público pelas três esferas de governo^10^.

Além da garantia esperada para os medicamentos na Rename, o Sistema Único de Saúde (SUS) estruturou sua assistência farmacêutica (AF) em torno de diversos programas e políticas para segmentos populacionais ou doenças específicas que incluem, de forma mais ou menos explícita, garantias distintivas de acesso a medicamentos. Mapear os segmentos em que há garantias específicas de acesso a medicamentos é um bom ponto de partida para monitorar o desempenho nessa área.

São escassos os estudos de abrangência nacional sobre a amplitude da cobertura farmacêutica no Brasil. Dados sobre compras públicas podem ser obtidos em registros administrativos do governo e usados para produzir informações sobre a disponibilidade de medicamentos. Mas há lacunas significativas nesses dados – em especial no que se refere às compras feitas por estados e municípios. Além disso, os dados não têm informações sobre o escopo e a cobertura populacional das políticas de AF.

Uma fonte relativamente pouco usada e que pode aumentar o detalhamento a esse respeito – por tipo de medicamento, de beneficiário e de consumo (aquisições com e sem desembolso direto) – é a Pesquisa de Orçamentos Familiares (POF)^11^. Além de informações sobre gastos, a POF pergunta aos entrevistados o valor estimado da aquisição de medicamentos obtidos sem desembolso direto – o que fornece informação potencial sobre financiamento público de medicamentos. A POF permite também conhecer a distribuição do consumo por faixas de renda e idade, tanto para as aquisições com quanto para as sem desembolso.

Assim, a partir de uma sistematização de programas e políticas que explicitem garantias específicas de AF no SUS, buscou-se identificar padrões de cobertura e consumo de medicamentos segundo faixas de renda e de idade daqueles que fazem aquisições sem desembolso direto de recursos. Entender quem se beneficia dessas aquisições é fundamental para monitorar e avaliar resultados de políticas nessa área.

## MÉTODOS

Trata-se de estudo observacional, usando dados da Pesquisa de Orçamentos Familiares (POF), do Instituto Brasileiro de Geografia e Estatística (IBGE). Inicialmente, foram pesquisadas no Sistema de Legislação da Saúde leis, portarias e normas relacionadas a programas que incluíssem como parte de seus objetivos o fornecimento de medicamentos. A partir dessa pesquisa, foram identificadas as principais garantias específicas de acesso com previsão de cobertura farmacêutica em vigor no SUS.

A POF é uma pesquisa domiciliar com amostra de 57.920 domicílios – selecionados em conglomerados nos diferentes estratos da pesquisa. Ela tem representatividade nacional e seu objetivo é descrever as estruturas de consumo e renda das famílias e também suas condições de vida. Com as variáveis do quadro 29 do questionário de despesas individuais da POF (Questionário 4), que discrimina 88 produtos farmacêuticos, foram elaborados tradutores para associar cada um dos programas previamente identificados aos tipos de medicamento da POF correspondentes. A seleção e compatibilização entre tipos de medicamento e programas de AF foi realizada por duas das autoras – uma médica e uma farmacêutica – com experiência em gestão de saúde pública.

Foi usada a edição mais recente da POF, apurada entre 11 de julho de 2017 e 9 de julho de 2018. Os dados sobre medicamentos na pesquisa se referem aos obtidos nos últimos 30 dias antes de cada entrevista.

Na pesquisa foram apurados os valores e a forma de aquisição de medicamentos (variável *V9002*), classificada como ‘com desembolso’ (aquisição monetária) e ‘sem desembolso’ (sem um pagamento direto de quem recebe o medicamento). No caso das aquisições sem desembolso, os entrevistados declararam valores estimados para os medicamentos obtidos. Os microdados da POF incluem também valores deflacionados para o período de referência de janeiro de 2018 (variável *V8000_defla*) para evitar que variações de preço ao longo dos meses da coleta de dados distorçam a interpretação dos resultados^11^.

Para cada tipo de medicamento listado na POF, foram agregados os valores e a quantidade de pessoas que reportaram aquisições com e sem desembolso segundo a faixa de idade (*V0403*) e a faixa de renda (variável *Renda_total* do domicílio de quem obteve o medicamento).

As agregações por idade abrangem 14 faixas entre 0–19 anos e 80 anos ou mais, com intervalos de cinco e de dez anos. As agregações por renda incluem sete faixas: até dois salários-mínimos (SM), 2–3 SM, 3–6 SM, 6–10 SM, 10–15 SM, 15–25 SM e > 25 SM.

Para verificar a consistência entre os valores estimados pelos entrevistados nas aquisições sem desembolso e os declarados nas aquisições com desembolso (*proxy* de preços de mercado) calculou-se, para cada tipo de medicamento, o valor médio mensal das aquisições com e sem desembolso de quem adquiriu o produto.

Uma análise descritiva, com estimativas pontuais e intervalares, foi realizada para: (a) valores médios mensais e percentuais de pessoas declarando consumo; (b) consumo total de medicamentos; (c) percentual de pessoas sem desembolso entre as que obtiveram medicamentos de programas com garantias específicas de acesso.

Cruzamentos de variáveis em que a frequência observada na amostra foi pequena foram excluídos das tabelas. As análises foram realizadas com o software *R* (versão 4.0.3) e o pacote *survey* (versão 4.0), que leva em conta o desenho do plano amostral da pesquisa. A participação das pessoas sem desembolso no total das que adquiriram medicamentos em cada grupo etário e de renda foi estimada com a função *svyciprop* para o cálculo do intervalo de confiança de 95%^12^.

## RESULTADOS

O Brasil tem vários programas ou políticas de saúde que envolvem garantias específicas de acesso a medicamentos. Para além das políticas mais abrangentes, PNM e PNAF, identificou-se as que têm relação com tipos específicos de medicamentos pesquisados na POF (Quadro).

Quando se considera o total de produtos farmacêuticos com dados apurados na POF e não apenas os medicamentos com garantias específicas de acesso no SUS, 63% das pessoas com renda domiciliar de até dois SM não consumiram medicamentos. Na faixa acima de 25 SM, esse percentual foi de 44,3% (Figura). O percentual de pessoas sem consumo diminui ao longo das faixas de renda, sugerindo que há restrições orçamentárias ao consumo nas faixas de renda mais baixas e/ou consumo excessivo nas faixas de renda mais altas.

**Figura:** Percentual de pessoas por forma de aquisição de medicamentos e por faixas de idade e renda no período de um mês. Brasil, 2017–2018.

Fonte: dados da Pesquisa de Orçamentos Familiares (POF), 2017–2018.

Pessoas com renda inferior a10 SM são as que mais têm consumo sem desembolso. Ainda assim, a proporção de aquisições sem desembolso é baixa em todas as faixas de renda, variando de 5,8% a 1,7% para as pessoas com aquisições apenas sem desembolso (Figura).

A partir da faixa de 2–3 SM, as aquisições sem desembolso – incluindo as pessoas com os dois tipos de aquisição no mês (categoria “Com e sem desembolso*”,* na figura) – diminuem com o aumento da renda. A menor proporção de pessoas com aquisições sem desembolso é a da faixa > 25 SM (4,5%). Na faixa até dois SM, esse percentual é de 9,9%.

Na análise por faixas etárias, o uso de medicamentos sem desembolso é mais expressivo entre os idosos, grupo em que grande parte das pessoas consome medicamentos de forma regular (39,2% dos usuários de medicamentos sem desembolso têm 60 anos ou mais). Na faixa etária de 70–74 anos, 77,3% das pessoas obtiveram medicamentos (com ou sem desembolso) no mês anterior à pesquisa.

Os medicamentos associados a programas de AF específicos responderam por 41,4% do valor do consumo total (com e sem desembolso) registrado na POF, mas concentram 67,8% do consumo sem desembolso de recursos. Do total de medicamentos com garantias específicas de acesso, 33,6% foram obtidos sem desembolso direto de recursos, ou seja, embora a maior parte dos medicamentos obtidos sem desembolso esteja associada a programas de AF, essas obtenções ainda representam apenas um terço do total para esses tipos de medicamento (uso sem desembolso como proporção do valor total das aquisições do medicamento).

Do total de produtos farmacêuticos listados na POF, 58,6% não está associado a nenhum programa ou política de saúde. Nesse grupo, 11,3% do valor foi de aquisições sem desembolso.

A Tabela 1 apresenta valores médios mensais de aquisições com e sem desembolso e o percentual do valor das aquisições e de pessoas com desembolso por tipo de medicamento da POF abrangido nas garantias específicas de acesso. Nas aquisições de medicamentos para hipertensão, colesterol, próstata e vias urinárias, o valor médio mensal de aquisições com desembolso foi maior do que para aquisições sem desembolso. Em medicamentos para problemas oculares ocorreu o oposto: o valor médio das aquisições sem desembolso foi maior. Para os outros produtos, as diferenças de preço (valor médio por aquisição) ficaram dentro dos intervalos de confiança.

**Tabela 1:** Valor médio mensal com e sem desembolso direto de recursos e percentual de valores e de pessoas que compraram medicamentos com previsão de garantias específicas no SUS (IC95%).

Medicamento	Valor de aquisição médio mensal com desembolso (R$)	Valor de aquisição médio mensal sem desembolso (R$)	Percentual do valor das aquisições com desembolso	Percentual de pessoas com desembolso
Vacinas	181,9 (89,5–274,3)	143,2 (109,0–177,4)	16,7 (6,5–26,9)	13,6 (7,4–19,8)
Para câncer	234,7 (160,5–308,8)	307,4 (174,6–440,2)	29,7 (17,1–42,4)	36,2 (24,9–47,5)
Para diabetes	96,1 (89,5–102,8)	70,9 (64,1–77,6)	52,1 (48,5–55,7)	45,8 (43,9–47,7)
Para doenças infecciosas e endêmicas	147,1 (124,3–169,9)	207,2 (162,6–251,8)	58,5 (46,9–70,0)	69,5 (60,6–78,4)
Para pressão alta (anti-hipertensivo)	47,9 (46,3–49,6)	34,8 (33,3–36,3)	64,1 (62,4–65,9)	57,7 (56,4–59,0)
Para redução de colesterol ou triglicerídeos	48,6 (46,2–51,0)	39,3 (35,1–43,5)	68,7 (65,2–72,2)	64,7 (62,3–67,0)
Para o sistema nervoso	117,9 (109,3–126,5)	116,8 (102,0–131,6)	70,0 (66,1–73,8)	72,6 (70,2–75,0)
Para asma e bronquite	93,5 (84,1–103,0)	98,8 (84,2–113,5)	70,8 (64,5–77,1)	74,1 (68,9–79,4)
Para depressão (antidepressivo)	110,7 (103,3–118,1)	95,2 (83,4–106,9)	74,3 (70,9–77,8)	72,6 (70,3–74,9)
Preservativo e lubrificante íntimo	15,6 (10,3–21,0)	13,8 (11,8–15,9)	75,3 (67,5–83,1)	73,3 (68,8–77,8)
Para estresse (calmante)	59,9 (56,2–63,7)	52,5 (44,0–60,9)	77,4 (74,0–80,9)	76,0 (73,6–78,4)
Para os ossos e articulações	97,8 (90,0–105,6)	93,4 (79,5–107,3)	81,8 (78,0–85,6)	82,3 (79,3–85,2)
Para problema ocular (oftalmológico)	65,2 (60,7–69,9)	89,6 (73,0–106,2)	86,0 (83,0–89,1)	90,7 (89,1–92,2)
Para problema ginecológico	58,6 (51,7–65,5)	54,3 (29,4–79,2)	88,4 (82,1–94,7)	87,6 (84,4–90,9)
Para próstata e vias urinárias	85,5 (78,8–92,2)	57,8 (46,2–69,5)	89,3 (85,6–93,0)	85,0 (80,6–89,4)
Anticoncepcional	23,5 (22,6–24,5)	20,2 (18,4–21,9)	90,3 (89,0–91,6)	89,0 (87,8–90,1)

Fonte: dados da Pesquisa de Orçamentos Familiares (POF), 2017–2018.

IC95% : intervalo de confiança de 95%.

Nota: medicamentos com amostra pequena ou resultados não significativos não foram incluídos.

Ordenando os medicamentos de acordo com o percentual das aquisições com desembolso no total, é possível ver uma relação entre esse percentual e o valor médio mensal por aquisição. O percentual de pessoas com desembolso entre as que obtiveram cada medicamento é, em geral, maior para os medicamentos com preços mais baixos.

Para o total dos produtos farmacêuticos listados na POF, 20,5% do valor foi de aquisições sem desembolso de recursos. Embora a maior parte do consumo de medicamentos no país dependa de desembolso direto das famílias, há itens para os quais a maior parte das aquisições ocorre sem desembolso. É o caso das vacinas (83,3% sem desembolso). As estimativas de valor das aquisições mensais e do número de pessoas que obtiveram medicamentos com e sem desembolso direto, por política e tipo de medicamento da POF, são apresentadas na Tabela 2.

**Tabela 2:** Consumo mensal de medicamentos com previsão de garantias específicas, em valor (R$ milhões) e quantidade de pessoas que os obtiveram (em milhares) com e sem desembolso direto (IC95%).

Políticas e programas / tipos de medicamentos da POF	Valores com desembolso	Valores sem desembolso	Pessoas com desembolso	Pessoas sem desembolso
Política Nacional de Atenção em Oftalmologia	**168,2 (151,7–184,7)**	**27,4 (20,9–33,8)**	**2.578,4 (2.397,8–2.759,0)**	**305,4 (250,3–360,6)**
Para problema ocular (oftalmológico)	168,2 (151,7–184,7)	27,4 (20,9–33,8)	2.578,4 (2.397,8–2.759,0)	305,4 (250,3–360,6)
Política Nacional de Atenção Integral à Saúde da Mulher	**192,0 (179,9–204,1)**	**21,6 (17,9–25,3)**	**7.138,0 (6.792,0–7.484,0)**	**910,2 (816,4–1.003,9)**
Anticoncepcional	153,9 (143,8–164,1)	16,6 (14,3–18,9)	6.543,2 (6.213,9–6.872,4)	821,4 (730,5–912,3)
Para problema ginecológico	38,1 (31,8–44,3)	5,0 (2,1–8,0)	650,1 (573,2–727,0)	92,5 (66,8–118,1)
Política Nacional de Atenção Oncológica	**18,8 (10,5–27,1)**	**44,4 (25,9–63,0)**	**80,1 (54,8–105,6)**	**144,6 (92,8–196,3)**
Para câncer	18,8 (10,5–27,1)	44,4 (25,9–63,0)	80,1 (54,8–105,5)	144,6 (92,8–196,3)
Política Nacional de Saúde Mental	**825,5 (777,0–874,1)**	**299,3 (268,0–330,6)**	**7.858,0 (7.527,0–8.190,0)**	**2.982,9 (2.792,5–3.173,4)**
Para depressão (antidepressivo)	331,1 (300,5–361,7)	114,3 (96,7–132,0)	2.991,4 (2.801,1–3.181,6)	1.201,3 (1.091,3–1.311,3)
Para estresse (calmante)	194,1 (176,7–211,6)	56,6 (45,3–67,8)	3.239,2 (3.038,9–3.439,6)	1.078,1 (957,9–1.198,4)
Para o sistema nervoso	299,1 (270,5–327,6)	128,4 (107,1–149,7)	2.537,3 (2.364,2–2.710,5)	1.099,6 (984,4–1.214,7)
Política Nacional de Atenção Integral da Saúde do Homem	**48,9 (42,1–55,8)**	**5,9 (3,8–8,0)**	**572,2 (501,5–642,9)**	**101,5 (69,0–134,0)**
Para próstata e vias urinárias	48,9 (42,1–55,8)	5,9 (3,8–8,0)	572,2 (501,5–642,9)	101,5 (69,0–134,0)
Programa Farmácia Popular do Brasil	**1.336,3 (1.275,0–1.397,5)**	**796,1 (748,2–844,0)**	**18.374,0 (17.841,0–18.908,0)**	**13.233,0 (12.775,5–13.690,5)**
Para asma e bronquite	60,8 (51,2–70,4)	25,1 (18,6–31,5)	650 (569,4–730,5)	253,5 (195,2–311,8)
Para diabetes	328,5 (298–359,1)	302,2 (268,4–336,0)	3.418 (3.225,7–3.610,3)	4.264 (4.043,3–4.484,7)
Para os ossos e articulações	123,2 (107,6–138,7)	27,5 (21,2–33,7)	1.260 (1.139–1.381)	294,1 (243,2–345)
Para pressão alta (anti-hipertensivo)	639,8 (609,5–670,1)	357,7 (336,2–379,2)	13.352,7 (12.923,5–13.781,9)	10.277,5 (9.874,6–10.680,4)
Para redução de colesterol ou triglicerídeos	184 (168,9–199,1)	83,7 (71,5–95,9)	3.782,8 (3.541,5–4.024,2)	2.129,3 (1.960,2–2.298,5)
Programa Nacional de DST/aids	**13,6 (8,6–18,6)**	**56,4 (24,8–88,0)**	**873,4 (740,4–1.006,5)**	**360,0 (293,9–426,1)**
Preservativo e lubrificante íntimo	13,6 (8,6–18,6)	4,5 (3,5–5,4)	873,4 (740,4–1.006,5)	323,3 (260,8–385,8)
Programa Nacional de Imunizações	**8,8 (3,0–14,5)**	**43,8 (28,8–58,7)**	**48,2 (24,6–71,8)**	**305,6 (231,3–379,9)**
Vacinas	8,8 (3,0–14,5)	43,8 (28,8–58,7)	48,2 (24,6–71,8)	305,6 (231,3–379,9)
Programas Estratégicos de Saúde	**29,6 (21,4–37,8)**	**21,1 (12,3–29,8)**	**201,3 (152,0–250,7)**	**101,6 (67,7–135,5)**
Para doenças infecciosas e endêmicas	29,6 (21,4–37,8)	21,1 (12,3–29,8)	201,3 (152,0–250,7)	101,6 (67,7–135,5)

Fonte: dados da Pesquisa de Orçamentos Familiares (POF), 2017–2018.

IC95%: intervalo de confiança de 95%; DST: doenças sexualmente transmissíveis.

Nota: medicamentos e programas com amostra pequena ou resultados não significativos não foram incluídos.

Além das vacinas, os medicamentos para câncer também foram, em sua maior parte, consumo sem desembolso (70,3%). Em seguida, destacam-se duas das três classes de medicamentos para doenças crônicas dispensadas em unidades do SUS e pelo Programa Farmácia Popular: medicamentos para diabetes e para hipertensão. A POF não tem dados separados para asma – o terceiro item com medicamentos sem copagamento no Programa Farmácia Popular. Entretanto os medicamentos “para asma e bronquite” na POF, têm participação de 29,2% para o valor do consumo sem desembolso.

No caso dos medicamentos para aids, a POF não apurou ocorrência de aquisição com desembolso, pois medicamentos para aids não são vendidos em farmácias comerciais. Assim, não há referência de preço para os usuários em suas estimativas de valor. Amostras pequenas no consumo de medicamentos para aids, autismo, alcoolismo e tabagismo e imunossupressores não permitiram uma estimativa robusta de médias mensais para esses itens.

Aquisições sem desembolso também tiveram participações expressivas em medicamentos para: doenças infecciosas ou endêmicas (41,5%), redução de colesterol (31,3%) e sistema nervoso (30%). Com percentuais menores para o valor do total de aquisições sem desembolso estão: anticoncepcionais (9,7%), medicamentos para próstata e vias urinárias (10,7%), medicamentos para problemas ginecológicos (11,6%) e medicamentos para problemas oftalmológicos (14%).

A Tabela 3 mostra o detalhamento, por faixas de idade e de renda, do percentual de pessoas sem desembolso entre as que obtiveram medicamentos relacionados a cada programa ou política de saúde.

**Tabela 3:** Percentual de pessoas sem desembolso direto entre as que obtiveram medicamentos com garantias específicas de acesso, por politica ou programa associado ao medicamento, faixa de idade e faixa de renda domiciliar (em salários mínimos) (IC95%).

Faixas de idade	Programa Nacional de DST/aids	Programa Farmácia Popular do Brasil	Programa Nacional de Imunizações	Política Nacional de Atenção em Oftalmologia	Política Nacional de Saúde Mental	Política Nacional de Atenção Integral à Saúde da Mulher
< 20	41,3 (28,2–55,7)	38,5 (26,6–51,9)	82,9 (61,9–93,5)	18,6 (7,5–39,1)	36,8 (28,4–46,1)	14,3 (10,9–18,5)
20–29	30,6 (23,6–38,6)	29,9 (24,2–36,2)	81,8 (63,0–92,2)	7,7 (3,2–17,1)	28,9 (23,0–35,5)	11,4 (9,7–13,4)
30–39	20,9 (15,0–28,3)	33,7 (30,1–37,6)	88,6 (74,4–95,4)	8,9 (4,5–16,7)	25,2 (21,7–29,0)	10,7 (8,9–12,8)
40–49	23,3 (14,8–34,7)	41,8 (39,3–44,3)	83,9 (64,1–93,8)	5,9 (3,3–10,2)	29,9 (26,6–33,5)	9,8 (7,5–12,7)
50–59	55,2 (33,4–75,2)	47,3 (45,2–49,3)	79,3 (54,2–92,6)	6,8 (4,5–10,3)	31,5 (28,5–34,6)	15,2 (9,7–23,1)
60–69	54,6 (22,9–82,9)	48,8 (46,9–50,8)	95,2 (69,5–99,4)	15,2 (10,9–20,7)	29,5 (25,9–33,3)	9,8 (2,7–30,1)
70–79	9,7 (0,2–82,8)	48,9 (46,6–51,2)	100,0 (ND)	15,4 (11,0–21,0)	26,6 (22,5–31,2)	25,1 (5,5–65,8)
> 79	ND	41,8 (38,3–45,4)	100,0 (ND)	9,5 (4,9–17,4)	18,9 (14,1–24,9)	22,0 (3,2–70,9)
**Faixas de renda**	**Programa Nacional de DST/aids**	**Programa Farmácia Popular do Brasil**	**Programa Nacional de Imunizações**	**Política Nacional de Atenção em Oftalmologia**	**Política Nacional de Saúde Mental**	**Política Nacional de Atenção Integral à Saúde da Mulher**
< 2	44,3 (34,2–54,9)	50,0 (47,8–52,1)	100,0 (ND)	15,1 (10,6–21,1)	33,8 (30,4–37,4)	16,0 (13,4–19,0)
2–3	34,6 (25,9–44,4)	50,7 (48,9–52,5)	94,5 (84,6–98,1)	13,2 (10,2–17,0)	34,9 (32,3–37,6)	12,1 (10,2–14,2)
3–6	31,7 (21,4–44,0)	51,5 (49,2–53,8)	88,6 (70,1–96,2)	11,4 (7,9–16,2)	36,4 (32,9–40,1)	15,4 (12,5–18,8)
6–10	13,6 (4,9–32,9)	30,7 (27,4–34,2)	74,3 (39,0–92,9)	4,5 (1,5–12,6)	12,4 (8,9–16,9)	5,6 (3,5–9,1)
10–15	20,4 (13,2–30,2)	43,3 (40,6–46,1)	82,8 (55,8–94,8)	8,3 (5,1–13,2)	23,4 (20,0–27,2)	8,1 (6,2–10,6)
15–25	8,4 (2,7–23,0)	24,9 (20,9–29,4)	52,1 (28,7–74,7)	4,1 (1,3–12,1)	9,8 (6,4–14,6)	5,7 (2,5–12,5)
> 25	6,5 (1,3–26,4)	13,5 (10,2–17,8)	65,8 (30,3–89,5)	3,8 (0,5–22,2)	4,4 (1,9–9,7)	4,1 (1,3–12,4)

Fonte: dados da Pesquisa de Orçamentos Familiares (POF), 2017–2018.

IC95%: intervalo de confiança de 95%; ND: não disponível; DST: doenças sexualmente transmissíveis.

Nota: programas com amostra pequena ou resultados não significativos não foram incluídos.

Na maioria dos programas e políticas com garantias específicas para medicamentos, as diferenças entre as faixas de idade não foram significativas. No caso de algumas políticas de saúde, o tamanho da amostra não permitiu a desagregação por faixas de idade e renda.

Exceto pela Política Nacional de Atenção em Oftalmologia (com intervalo de confiança muito largo para permitir essa conclusão), para os programas estudados, as faixas de zero a 15 salários mínimos tiveram percentuais mais altos de pessoas que obtiveram medicamentos sem desembolso. As pessoas em domicílios com renda acima de 15 salários mínimos formam um outro grupo, com menor participação das que obtiveram medicamentos sem desembolso (Tabela 3).

## DISCUSSÃO

A POF mostra que 20,5% do consumo de medicamentos (em valor) ocorreu sem desembolso das famílias. A maior parte das aquisições de medicamentos sem desembolso foi feita por pessoas nas faixas de renda domiciliar até 10 salários mínimos e nas faixas etárias mais altas. Ainda assim, a participação do financiamento público no consumo de medicamentos é notoriamente baixa no Brasil e está longe da média da OECD, de 58%^23^.

Na Conta-Satélite de Saúde^2^, publicação do IBGE com dados agregados sobre bens e serviços relacionados à saúde, o consumo de medicamentos sem desembolso direto das famílias brasileiras representa 7,5% do consumo total. A principal razão para a diferença entre os percentuais sem desembolso na POF e na Conta-Satélite tem relação com preços e com o tipo de medicamento comprado pelo governo. O governo disponibiliza medicamentos com base em prescrições médicas e uso racional e paga preços menores do que os praticados para venda às famílias, pois conta com os Preços Máximos de Venda Governamental, abaixo dos de mercado para alguns medicamentos, e compra em escalas maiores. Assim, para um mesmo total de recursos, o governo compra mais unidades de medicamentos do que as famílias. É possível dizer que, estimado com preços similares para as aquisições com e sem desembolso, o percentual sem desembolso da POF está mais perto de descrever a proporção dessas aquisições em termos de quantidades físicas do que a comparação de valores feita na Conta-Satélite^24^.

As garantias específicas contidas em determinadas políticas e programas específicos (como o Programa Nacional de Imunizações e o Programa Farmácia Popular) parecem ter assegurado um acesso sem desembolso superior ao da lista de garantias gerais da PNAF. Isso configura um padrão semelhante ao de países em desenvolvimento, que tendem a concentrar o financiamento público da AF em nichos específicos de populações ou doenças^4^.

Entretanto, as garantias específicas de acesso a medicamentos têm coberturas muito distintas. Entre os medicamentos analisados com maior participação do consumo sem desembolso estão as vacinas (83,3%) e os medicamentos para câncer (70,3%). No outro extremo, com baixo percentual de aquisições sem desembolso, estão anticoncepcionais (9,7%), medicamentos para próstata e vias urinárias (10,7%), para problemas ginecológicos (11,6%) e para problemas oftalmológicos (14%).

O acesso sem desembolso a medicamentos para hipertensão e diabetes via Programa Farmácia Popular evitou internações no SUS e mortes relacionadas a essas doenças nos municípios em que o programa foi implementado^25^. Quando esses medicamentos passaram a ter distribuição gratuita, sem copagamento, seu uso aumentou, indicando que, para muitas pessoas, o preço é uma barreira ao acesso, ainda que esses produtos sejam dispensados em farmácias das unidades de saúde do SUS^26,27^.

Pessoas com menor renda obtêm medicamentos sem desembolso com mais frequência que pessoas nas faixas mais altas. Como, para elas, o preço é mais frequentemente uma barreira ao acesso^26^, a obtenção sem desembolso contribui para evitar a piora de seu estado de saúde, possíveis internações e também mortes precoces por interrupção do tratamento de doenças crônicas já diagnosticadas^25^.

Depreende-se que a redução do financiamento público de medicamentos, observada em registros administrativos desde 2016, pode ter efeitos graves não só sobre a saúde da população como sobre as despesas com serviços – sobrecarregando a rede hospitalar com casos evitáveis^25^.

O programa de AF de menor cobertura foi o de atenção à mulher. Esse foi um achado surpreendente considerando o destaque desse segmento de políticas – relacionadas às metas três e cinco dos Objetivos de Desenvolvimento Sustentável (ODS)^28^. As políticas de atenção à saúde do homem, atenção em oftalmologia e controle do tabagismo também tiveram baixa participação de medicamentos obtidos sem desembolso direto. Entre as políticas mais recentes, o baixo desembolso exigido para acesso a alguns medicamentos, como os anticoncepcionais, vendidos a preços mais baixos em farmácias privadas, pode ser um dos motivos da baixa cobertura nesses segmentos. O incentivo para buscar medicamentos públicos está provavelmente ligado aos altos preços e às baixas barreiras de acesso. Procedimentos complexos para obter os medicamentos podem desmotivar essa procura, penalizando em especial aqueles com menor renda. Isso sugere a necessidade de simplificar procedimentos de acesso a medicamentos em que a obtenção sem desembolso tem sido baixa, como no caso de anticoncepcionais.

Chama atenção a boa cobertura nos chamados “medicamentos da pobreza” (vacinas e para doenças endêmicas). Esses medicamentos têm foco em doenças transmissíveis e figuram entre as mais antigas garantias específicas na AF, com o Programa Nacional de Imunização precedendo inclusive à PNAF^29^.

Este estudo tem algumas limitações. Para idades muito avançadas, a amostra pequena conduz a estimativas pouco robustas, dada a variabilidade dos dados. No caso de medicamentos menos usados (como os para tabagismo ou para câncer), amostras pequenas produzem variâncias grandes impedindo uma análise significativa por faixa de idade ou de renda.

Há diferenças entre os valores apresentados na POF para obtenção de medicamentos sem desembolso e os registros administrativos do governo para despesas com programas específicos – como o para aids. Essa limitação da base de dados está relacionada à atribuição de valores, pelos entrevistados na POF, para os medicamentos obtidos sem desembolso e também ao tamanho da amostra no caso de medicamentos usados por uma parte pequena da população.

A pesquisa não registra aquisições de medicamentos por pessoas com menos de 10 anos de idade. Como o registro está associado a quem fez a aquisição (e não a quem usou o medicamento), pode-se supor que a aquisição para pessoas na faixa mais baixa é, em grande parte, feita por seus pais e registrada como deles, levando à possível superestimativa no consumo das faixas etárias com filhos ou dependentes menores.

Merecem também menção a distribuição gratuita de medicamentos vinculada à amostra grátis^30^e, desde 2014, a distribuição de antineoplásicos orais e medicamentos para combate a efeitos colaterais da quimioterapia por planos de saúde^31^, que podem responder por parte do consumo sem desembolso. Seria incorreto afirmar que toda a aquisição de medicamentos sem desembolso na POF está relacionada a financiamentos do SUS.

O fato de os valores do consumo sem desembolso (aquisições não monetárias, na POF) serem estimados pelos declarantes tem motivado desconfiança em relação a esses dados e poderia ser considerado uma limitação. Entretanto, comparando valores médios mensais para aquisições com e sem desembolso para os tipos de medicamentos estudados, não foram encontradas diferenças substanciais – o que justifica o uso desses dados. O uso dos dados de aquisições não monetárias é uma das contribuições positivas deste artigo para o estudo da cobertura da AF.

As políticas e programas analisados receberam destaque neste estudo porque têm previsão de oferta de medicamentos de forma específica para seus beneficiários. Mas é importante lembrar que o direito à assistência terapêutica integral é componente do direito à saúde e que, para garanti-lo, os entes da Federação têm implementado uma série de medidas para o fortalecimento da AF no SUS. Uma ampla rede de farmácias públicas é responsável por sua dispensação em todo o país. Contudo, a baixa disponibilidade de medicamentos nessas farmácias^32^ pode explicar, em parte, os percentuais elevados de aquisição de medicamentos com desembolso direto, mesmo para aqueles que têm garantias extras definidas em políticas e programas específicos e para tratamento de doenças de alta prevalência. Essa situação indica a existência de barreiras de acesso a esses produtos no SUS.

Os resultados deste artigo sugerem a necessidade de fortalecer e ampliar as políticas de AF, que têm beneficiado principalmente pessoas de menor renda ou maior consumo relacionado à idade. Os dados sobre aquisições monetárias e não monetárias de medicamentos da POF, ainda que de maneira indireta, ajudam a descrever o alcance dessas políticas no que se refere ao acesso a medicamentos.

**Quadro:** Garantias específicas de acesso a medicamentos em políticas e programas de saúde, segundo tipos de medicamento da POF.

Políticas/Programas	Garantias específicas de acesso a medicamentos	Tipos de medicamentos da POF
Política Nacional de Atenção em Oftalmologia	Assistência farmacêutica no SUS como elemento da política, com foco no tratamento do glaucoma. Inclusão de medicamentos no Componente Especializado da Assistência Farmacêutica (CEAF)^13,14^.	Para problema ocular (oftalmológico)
Política Nacional de Atenção Integral à Saúde da Mulher	Oferta de métodos anticoncepcionais para a população em idade reprodutiva. Inclui contraceptivos orais e injetáveis, dispositivo intrauterino (DIU) e diafragma^13,14^.	Anticoncepcional
		Para problema ginecológico
Política Nacional para a Prevenção e Controle do Câncer na Rede de Atenção à Saúde das Pessoas com Doenças Crônicas	Acesso dos pacientes à quimioterapia. Com o lançamento de quimioterápicos de uso oral, os centros de alta complexidade em oncologia (CACON) e as unidades de assistência em alta complexidade em oncologia (UNACON) passaram a dispensar medicamentos quimioterápicos para uso pelos pacientes em seus domicílios. O Ministério da Saúde realiza a compra centralizada de alguns antineoplásicos com o objetivo de reduzir o custo dos tratamentos no SUS e aumentar o acesso da população ao tratamento^13,15^.	Para câncer
Política de Saúde Mental	Acesso a medicamentos para pessoas com sofrimento ou transtorno mental. Na Relação Nacional de Medicamentos Essenciais (Rename) estão incluídos medicamentos para tratamento de diversos problemas de saúde nessa área: ansiolíticos, antidepressivos, antipsicóticos, entre outros^13,14,16^.	Para autismo
		Para depressão (antidepressivo)
		Para estresse (calmante)
		Para o sistema nervoso
Política Nacional de Transplantes de Órgãos e Tecidos	Não existe uma política formal com essa denominação. Mas, sim, um Sistema Nacional de Transplantes, além de protocolos clínicos e diretrizes terapêuticas que estabelecem o uso de imunossupressores e de outros medicamentos no tratamento dos pacientes transplantados. Os medicamentos constantes dos protocolos estão incluídos na Rename^14,17^.	Medicamentos imunossupressores
Política Nacional de Atenção Integral da Saúde do Homem	Prevê entre as suas diretrizes o tratamento dos agravos e enfermidades do homem. Na Rename, estão incluídos medicamentos para o tratamento da hiperplasia prostática benigna^13,14^.	Para próstata e vias urinárias
Programa Farmácia Popular do Brasil	Dispensação por farmácias privadas, mediante copagamento dos usuários, de uma lista de medicamentos usados no tratamento da dislipidemia, osteoporose, glaucoma, rinite e doença de Parkinson, além de contraceptivos e fraldas geriátricas. Dispensação gratuita ao paciente de medicamentos usados no tratamento da hipertensão, diabetes e asma^18,19^.	Para asma e bronquite
		Para diabetes
		Para os ossos e articulações
		Para pressão alta (anti-hipertensivo)
		Para redução de colesterol ou triglicerídeos
Programa Nacional de Controle do Tabagismo	Acesso a medicamentos para tratamento das pessoas tabagistas^14,16^.	Combate ao alcoolismo e tabagismo
Programa Nacional de DST/aids	Acesso a medicamentos antirretrovirais e a outros medicamentos para tratamento de doenças oportunistas^14,20^.	Para aids
		Preservativo e lubrificante íntimo
Programa Nacional de Imunizações	Acesso a vacinas e soros^14,21^.	Vacinas
Programas Estratégicos de Saúde	Não existe um programa formal com essa denominação. Agrega um conjunto de programas em saúde, tais como controle da tuberculose, da hanseníase, das endemias focais, da gripe (Influenza), além da prevenção de deficiências nutricionais e do programa de sangue e hemoderivados^14,22^.	Para doenças infecciosas e endêmicas

POF: Pesquisa de Orçamentos Familiares; DST: doenças sexualmente transmissíveis.
